# Doxorubicin inhibits miR-140 expression and upregulates PD-L1 expression in HCT116 cells, opposite to its effects on MDA-MB-231 cells

**DOI:** 10.3906/biy-1909-12

**Published:** 2020-02-17

**Authors:** Noel Mopouguini NABA, Nazife TOLAY, Batu ERMAN, Ayca SAYI YAZGAN

**Affiliations:** 1 Department of Molecular Biology and Genetics, Faculty of Science and Letters, İstanbul Technical University, Maslak, İstanbul Turkey; 2 Molecular Biology, Genetics, and Bioengineering Program, Faculty of Engineering and Natural Sciences,Sabancı University, Tuzla, İstanbul Turkey; 3 SUNUM Nanotechnology Research Center, Sabancı University, İstanbul Turkey

**Keywords:** PD-L1, doxorubicin, miR-34a, miR-140

## Abstract

One of the most challenging problems in colorectal cancer (CRC) is resistance to chemotherapy drugs such as doxorubicin (DOX). The programmed death ligand-1 (PD-L1) is related to chemoresistance and is overexpressed in several human cancer cell types. Here, we investigated the changes in the expression of PD-L1 in DOX-treated CRC and breast cancer (BRC) cells. Also, to address PD-L1 regulation, we assessed expression levels of miR-140 and miR-34a, two microRNAs that can target the 3’ UTR region of the gene encoding PD-L1. HCT116 CRC and MDA-MB-231 BRC cells were treated with various doses of DOX in culture and PD-L1 expression was quantified using qRT-PCR, flow cytometry, and western blot analysis. We also evaluated PD-L1 localization in HCT116 cells by immunofluorescence. Next, we assessed expression of miR-140 and miR-34a in DOX-treated HCT116 and MDA-MB-231 cells. Finally, we investigated whether miR-140 targets the 3’ UTR of the gene encoding PD-L1 in HCT116 cells using the p2FP-RNAi RNAi reporter vector system. PD-L1 expression in HCT116 cells, while low at baseline, can be induced by treatment with 0.5 µM DOX. MDA-MB-231 baseline PD-L1 expression exceeded HCT116 cell maximal expression and decreased following DOX treatment. We further demonstrated that PD-L1 localizes to the cell surface in DOX-treated HCT116 cells. While miR-140 expression decreased in DOX-treated HCT116 cells, it increased in DOX-treated MDA-MB-231 cells. MiR-34a expression increased in both DOX-treated cell types. Finally, we present evidence for the regulation of PD-L1 by miR-140 in HCT116 cells. PD-L1 expression can increase following treatment with DOX in HCT116 cells but decrease in MDA-MB-231 cells, suggesting a distinct response to DOX in these two different cancer types. Also, a negative correlation between PD-L1 and miR-140 was observed in DOX-treated HCT116 cells, but not in MDA-MB-231 cells.

## 1. Introduction

Colorectal cancer is reported as the leading cause of cancer-related death worldwide (Torre et al., 2012). Most patients with CRC are diagnosed in advanced stages, where the main option for the treatment is chemotherapy (Moertel et al., 1994). Chemotherapeutic drugs such as 5-fluorouracil and oxaliplatin are used in CRC treatment despite their severe side effects like risk of infection, anemia, and peripheral neuropathy (Midgley et al., 2005). Therefore, alternative chemotherapeutic drugs would be beneficial for patients. 

Doxorubicin (DOX), which belongs to the anthracycline family, is used for the treatment of various cancer types including breast cancer, lung cancer, gastric cancer, and leukemia, but not for CRC (Minotti et al., 2004). Even though DOX in high doses has been shown to be an efficient chemotherapeutic drug for the treatment of advanced stage CRC, it is not the drug of choice because high-dose treatment has side effects such as severe cardiotoxicity (Ewer et al., 2015). 

Programmed death ligand-1 (PD-L1, CD274, and B7-H1), which is a transmembrane glycoprotein expressed mainly on antigen presenting cells (APCs) and tumor cells, inhibits activated T-cells by binding to their inhibitory receptor, PD-1, in the tumor microenvironment (Tamura et al., 2001; Selenko-Gebauer et al., 2003; Zang et al., 2007). PD-L1 expression in multiple solid tumors, including CRC, was shown to be associated with worse survival (Wu et al., 2015). Recently developed immunotherapies, which aim to block the PD-1/PD-L1 pathway using specific monoclonal antibodies, dramatically improve antitumor immune responses in various cancer types like skin cancer and non-small cell lung cancer (Zang and Allison, 2007; Topalian et al., 2012). The PD-1/PD-L1 interaction was shown to increase resistance of prostate and breast cancer cells to DOX in vitro. Also, inhibition of PD-1/PD-L1 using antibodies against PD-1 improved the effect of DOX to reduce metastasis in an in vivo model of breast cancer (Black et al., 2016).

MicroRNAs (miRNAs) are short (20–22 nt) noncoding RNAs that regulate gene expression at the posttranscriptional level. Aberrant expression of miRNAs is linked to many human disorders including cancer (Volinia et al., 2006). PD-L1 expression is regulated by various miRNAs in different cancer types (Wang et al., 2017). miR-34a was reported to target PD-L1 in various cancer cells including cells of osteosarcoma, acute myeloid leukemia, and lung, breast, and colon cancer (Cortez et al., 2015; Wang et al., 2015). Also, miR-140 targets PD-L1 in osteosarcoma and lung cancer (Ji et al., 2018; Xie et al., 2018). DOX treatment can upregulate miR-140 expression in osteosarcoma cells and miR-34a in lymphoma patients (Wei et al., 2016; Zhu et al., 2017). These findings show that control of PD-L1 expression may be complex and tissue-specific. In this light, the effect of DOX on PD-L1 expression and microRNAs (miR-34a and miR-140) in CRC cells has not been investigated. 

Here, we evaluate the levels of PD-L1 and microRNAs (miR-140-3p and miR-34a) expressions in DOX-treated HCT116 colon cancer and MDA-MB-231 breast cancer cell lines. We found that DOX treatment increased PD-L1 expression in both the mRNA and protein levels in HCT116 cells, but not in MDA-MB-231 cells. Also, we demonstrate that miR-140-3p but not miR-34a expression is downregulated in DOX-treated HCT116 cells, resulting in increased PD-L1 expression.

## 2. Materials and methods

### 2.1. Cell culture

HCT116 cells were obtained from the ATCC (USA). MDA-MB-231 cells were provided by Dr. Gizem Dinler Doğanay (İstanbul Technical University, İstanbul, Turkey). Cells were kept in DMEM medium (Lonza, Germany) including 10% fetal calf serum (FCS) (Invitrogen Corp., UK), penicillin, and streptomycin (Invitrogen Corp., UK). Cell lines were assessed to evaluate mycoplasma contamination using the Mycoplasma Detection Kit (Life Technologies). Cells were maintained at 37 °C in a humidified 5% CO2 incubator.

### 2.2. Doxorubicin treatment

Twenty-four hours after seeding, cells were incubated with different concentrations of DOX (Sigma-Aldrich, USA), solubilized in DMSO, for 18 h.

### 2.3. Staining and flow cytometry

For surface staining, cells were stained with antihuman CD274 (B7-H1, PD-L1)-APC (clone 29E.2A3) antibody (BioLegend, USA) in PBS containing 2% FCS for 1 h. Flow acquisition was done with a BD Accuri C6 Cytometer (Becton Dickenson, USA). Flow cytometric analyses were performed using FlowJo Software (Tree Star, USA).

### 2.4. Western blot analysis

Cell pellets were lysed in a whole cell extract lysis (WCE) buffer that includes 1 M HEPES, 0.4 mM NaCl, 25% glycerol, 1 mM EDTA, 15 mM NaF, and 0.1% NP-40, supplemented with protease inhibitors (Thermo Scientific) and phosphatase inhibitors (Roche PhosSTOP) for 30 min at 4 °C. Cell lysates were assayed for protein concentration using the BCA protein assay (Thermo Fisher Scientific, USA). Whole cell extracts were subjected to 10% SDS-PAGE and transferred to nitrocellulose membranes for immunoblotting. Ponceau S staining was performed to validate efficient transfer. Membranes were placed together with the following primary antibodies: antihuman PD-L1 (1:100; clone 28-8, Abcam) and antihuman vinculin (1:1000; clone EPR 8185, Abcam). Binding of primary Abs was detected with HRP-conjugated antirabbit (clone Ab 205718, Abcam) (1:1000). LumiGLO reagent (Cell Signaling Technology) was used for enhanced chemiluminescence detection. The blots were visualized using a chemiluminescent western blotting detection system (ChemiDoc MP imaging system; Bio-Rad) and analyzed by Bio-Rad Image Lab software.

### 2.5. Immunofluorescence staining

HCT116 cells were plated on poly-L-lysine-coated coverslips at 70% confluency. Cells were treated with 0.5 µM DOX for 18 h. After the treatment, samples were placed in methanol for 20 min at –20 °C. Later, coverslips were rinsed with PBS and incubated in blocking buffer for 30 min at RT and with primary mouse antihuman PD-L1 antibody (1:50; clone 22C3, Dako) o/n at 4 °C. After washing with PBS, coverslips were incubated together with goat antimouse IgG (Alexa Fluor 594) secondary antibody (1:100) (Cell Signaling Technologies) for 1 h at RT. Coverslips were mounted in mounting medium (VECTOR Laboratories Inc.). Image acquisition was performed using a LSM 710 confocal microscope (Carl Zeiss Micro Imaging) using the 405 nm and 561 nm laser lines to excite DAPI and Alexa Fluor 594, respectively. 

### 2.6. RNA extraction and q-RT-PCR

Total RNA was isolated from HCT116 and MDA-MB-231 cells using the Quick-RNA

Miniprep Plus Kit (Zymo Research). cDNA was synthesized by a high-capacity cDNA synthesis kit (Applied Biosystems). Q-RT-PCR was done on the ABI StepOnePlus system (Applied Biosystems). 18S rRNA expression was used for normalization of gene expression of each sample. Mean relative gene expression was calculated. Also, the differences were determined using the method of 2–ΔΔCT in triplicate analyses. Primer sequences were PD-L1 forward 5’- AAGACCACCACCACCAATTC -3’, reverse PD-L1 5’- CTGGGATGACCAATTCAGC -3’, 18S rRNA forward 5’-GGCCCTGTAATTGGAATGAGTC -3’, and reverse 5’ – CCAAGATCCAACTACGAGCTT- 3’. 

For the expression analysis of miRNAs, total RNA including miRNA was extracted using the Quick-RNA Miniprep Plus Kit (Zymo Research, USA). Both cDNA synthesis and miRNA amplification and detection were performed using the EPIK miRNA Select Hi/Lo-ROX Kit (Bioline, UK). The q-RT-PCR assay was done by StepOnePlus Real-Time PCR System (Applied Biosystems). The relative miRNA expression was calculated through the comparative CT method. The CT value of the target gene was normalized by the U6 housekeeping reference. Primers for miR-140-3p (MIMAT0004597), miR-34a-5p (MIMAT0000255), and the U6 housekeeping gene (BIO66046) were purchased from Bioline (UK). All reactions were performed in triplicate. Comparison between DOX-treated and untreated cell lines was done using the unpaired Student t-test (P < 0.05 significant).

### 2.7. miRNA mimics and reporter vector transfection

Reporter plasmids containing the PD-L1 gene 3’ UTR (containing the miR140 target site) were generated by PCR amplification of genomic DNA extracted from HCT-116 cells using the primers PDL-FWD: 5’-GGA**AGATCT**TCCAGCATTGGAACTTCTGATCTTC-3’ and PDL-REV: 5’-CCC**AAGCTT**TGCCTGGCACAGCGATTGATATTG-3’ containing restriction sites for the Bgl II and Hind III restriction enzymes, respectively (bold letters). A PCR fragment of 1048 bp was digested with Bgl II and Hind III and cloned into the same sites of the multiple cloning site of the p2FP-RNAi dual color vector (Evrogen, FP981) immediately after the coding region of the JRed fluorescent protein gene.

### 2.8. Fluorescent miRNA activity assay

HCT116 cells were kept in DMEM with 10% FCS, penicillin, and streptomycin. Cells were cultured 24 h before treatment at 40% confluency in 6-well plates (300,000 cells/well). Prior to the transfection, growth medium was changed with 2 mL of fresh medium. Cells were transfected by preparing a mixture containing 2 µg of p2FP-RNAi plasmid DNA and 6 µL of 1 µg/µL polyethyleneimine, linear, MW 25,000 (Polysciences, 23966) in 100 µL of medium without serum, antibiotics, or phenol red. For assessing the activity of miRNA mimics, a second mixture containing either 1 µL of nontargeting mimic or miR-140 mimic and 6 µL of 1 µg/µL PEI in 100 µL of DMEM was simultaneously added to HCT116 cells. Later, cells were analyzed by flow cytometry after 48 h. Flow cytometry was performed on a FACS Fortessa flow cytometer with appropriate filters to detect TurboGFP fluorescence (excitation: 482 nm; emission: 538 nm) or JRed fluorescence (excitation: 546 nm; emission: 607 nm) using 488 nm and 561 nm lasers to excite TurboGFP and JRed, respectively. We calculated net fluorescence by dividing the mean fluorescence of the empty p2FP-RNAi plasmid transfected cells by that of PD-L1 3’ UTR containing p2FP-RNAi plasmid-transfected cells. The GFP mean fluorescence intensities of either control mimic or miR-140 mimic expressing PD-L1 3’ UTR containing p2FP-RNAi plasmid transfected cells were divided by their JRed mean fluorescence intensity to calculate a GFP/JRed mean ratio. Results are representative of two experiments. 

### 2.9. Bioinformatics analysis

The TargetScan web server was used to predict target miRNA binding to the 3’ UTR region of PD-L1.

### 2.10. Statistical analysis

Data are presented as mean ± SEM. Unless otherwise stated, the unpaired Student t-test was used for comparison. Graphs were prepared using GraphPad Prism software. Pearson correlation was used to evaluate potential correlation between miR-140-3p and PD-L1. P < 0.05 shows statistical significance.

## 3. Results

### 3.1. DOX induces PD-L1 expression in HCT116 colon cancer cells 

**Figure 1:**
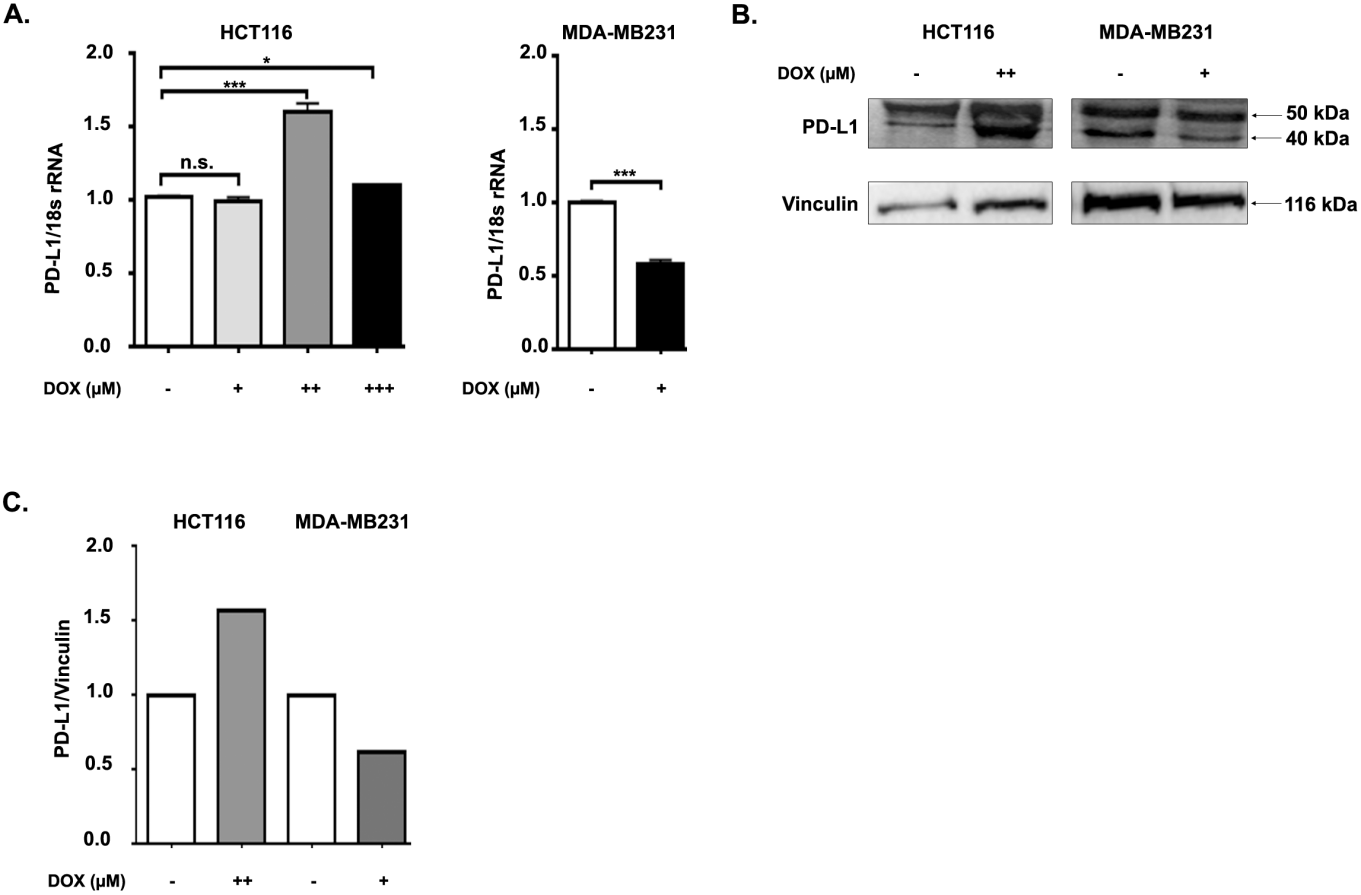
Doxorubicin regulates PD-L1 mRNA and protein in HCT116 and MDA-MB-231 cells in an opposite way. HCT116 and MDA- MB-231 cells were incubated with various concentrations of doxorubicin (DOX) (for HCT116 cells (+) 0.1 ?M, (++) 0.5 ?M, (+++) 1.5 ?M; for MDA-MB-231 cells (+) 0.7 ?M) or without any treatment for 18 h. A) The mRNA expression of PD-L1 in DOX-treated or nontreated HCT116 and MDA-MB-231 cells was assessed by qRT-PCR. Data are normalized to 18S rRNA and are representative of 3 independent experiments. P-values were calculated via unpaired t-test (**P < 0.01; ***P < 0.001). B) Protein expression of PD-L1 in DOX-treated or nontreated HCT116 and MDA-MB-231 cells evaluated by immunoblotting. Vinculin was used as a loading control. Immunoblots shown are representative of 3 independent experiments. n.s. denotes a nonspecific band. C) Densitometric quantification of PD-L1 protein expression levels in DOX-treated and nontreated HCT116 and MDA-MB-231 cells was performed by normalizing to vinculin.

In order to examine whether doxorubicin has an effect on PD-L1 expression in CRC cell lines, we treated the HCT116 CRC cell line with 0.1, 0.5, and 1.5 µM DOX for 18 h. It was reported that DOX has a suppressive effect on cell surface expression of PD-L1 in MDA-MB-231 cells in a dose-dependent manner (Ghebeh et al., 2010). We treated MDA-MB-231 cells with a dose (0.7 µM) of DOX that was shown to cause 50%–60% decrease in surface PD-L1 expression (Ghebeh et al., 2010). Next, we assessed both mRNA and protein expression levels of PD-L1 in DOX-treated HCT116 and MDA-MB-231 cells. Interestingly, we detected by qRT-PCR that 0.5 µM and to a lesser extent 1.5 µM, but not 0.1 µM DOX, induced PD-L1 mRNA expression in HCT116 cells (Figure 1A). In contrast, PD-L1 mRNA expression in MDA-MB-231 cells was reduced significantly by DOX treatment, which confirmed the findings in the literature (Figure 1A). 

Next, we evaluated total PD-L1 protein levels in DOX-treated HCT116 and MDA-MB-231 cells by western blotting. We selected DOX concentrations that led to an effect on PD-L1 mRNA expression in HCT116 and MDA-MB-231 cells, 0.5 µM and 0.7 µM doxorubicin, respectively. PD-L1 has been reported to include four N-glycosylation sites, three of which are related to stabilization (Li et al., 2016). Therefore, there is a heterogeneous pattern of PD-L1 on western blotting with glycosylated bands of about 40–50 kDa. The nonglycosylated PD-L1 is 33 KDa. While DOX treatment resulted in increased PD-L1 protein expression in HCT116 cells, it decreased in MDA-MB-231 cells (Figures 1B and 1C). Overall, these data indicate that DOX treatment affects BCC and CRC cells differently, causing a decrease in MDA-MB-231 cells and an increase of PD-L1 expression in HCT116 CRC cells both in RNA and protein levels. 

### 3.2. PD-L1 localizes to the cytoplasm and cell membrane in DOX-treated HCT116 cells

**Figure 2:**
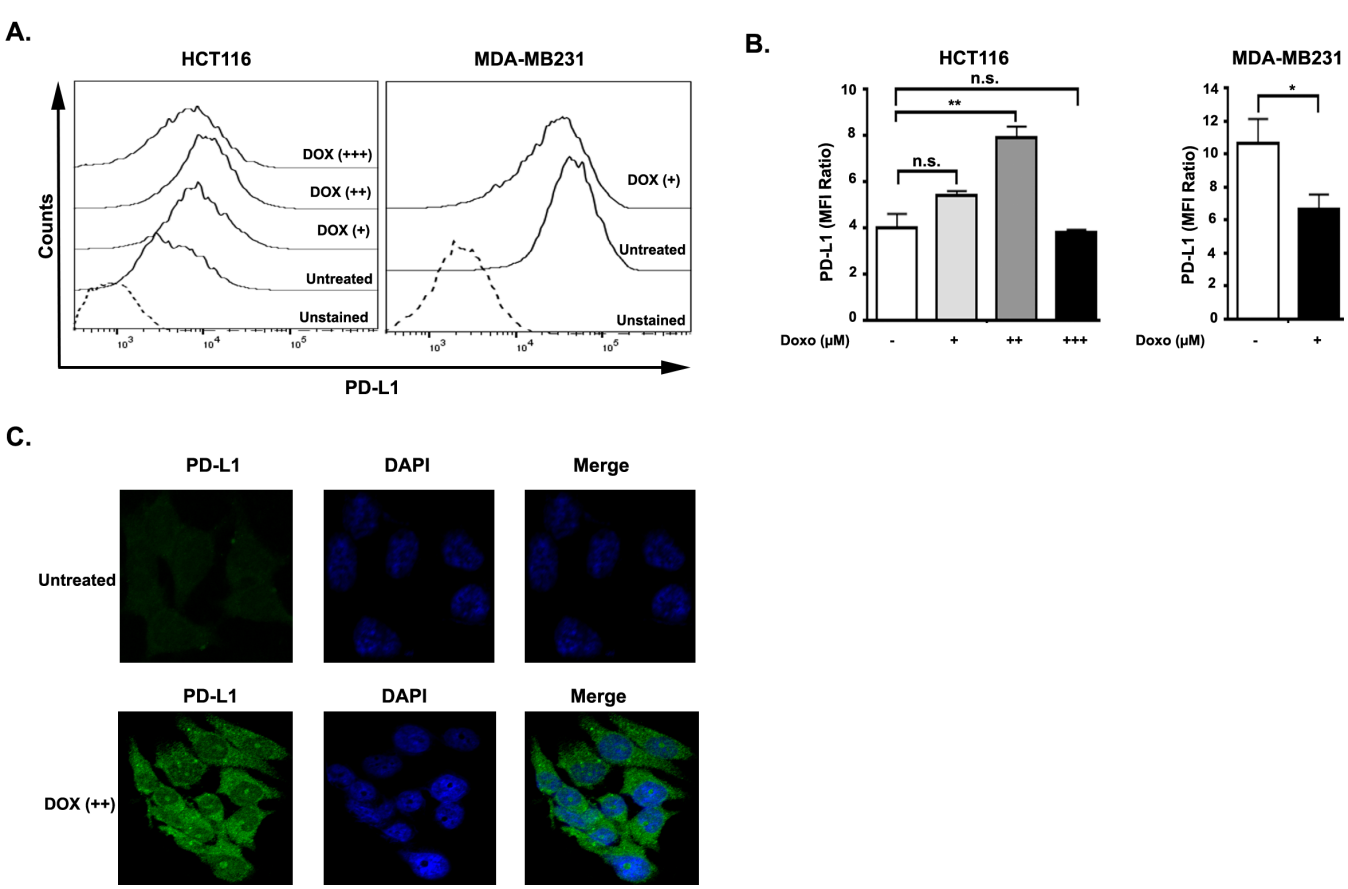
PD-L1 surface expression was regulated conversely in HCT116 and MDA-MB-231 cells by doxorubicin treatment. A) Representative histogram of surface expression of PD-L1 on HCT116 and MDA-MB-231 cells after treatment with different concentrations of DOX (for HCT116 cells (+) 0.1 ?M, (++) 0.5 ?M, (+++) 1.5 ?M; for MDA-MB-231 cells (+) 0.7 ?M) for 18 h. B) Flow cytometric measurement of three independent experiments are represented as MFI ratio = (MFIPD-L1/MFIunstained) SEM. Significance was calculated using one-way analysis of variance (ANOVA). P-values are as follows: *P < 0.05; **P < 0.01. C) Representative immunofluorescence image of HCT116 cells after 0.5 ?M DOX treatment or kept untreated for 18 h. Anti-PD-L1 antibody is used to stain PD-L1 (green) and DAPI (blue) is used to stain nucleus.

Recent studies demonstrated that DOX downregulates surface PD-L1 expression in MDA-MB-231 breast cancer cells in a dose-dependent manner (Ghebeh et al., 2010). In order to investigate the level of surface PD-L1 expression in HCT116 and MDA-MB-231 cells, we treated these cells with DOX for 18 h and analyzed surface expression by flow cytometry. As expected, 0.7 µM DOX treatment of MDA-MB-231 cells resulted in a significant decrease in surface expression of PD-L1, which is consistent with the previous literature (Figures 2A and 2B) (Ghebeh et al., 2010). Strikingly, we found that 0.5 µM DOX treatment caused a pronounced induction rather than a decrease of surface PD-L1 expression in HCT116 cells. Also, 0.1 µM DOX resulted in an increase of surface PD-L1 expression in these cells. However, no significant induction of PD-L1 expression was shown in 1.5 µM DOX-treated HCT116 cells (Figures 2A and 2B).

Next, we evaluated the cellular localization and level of PD-L1 expression in DOX-treated and nontreated HCT116 cells by immunofluorescence. Previous studies reported anomalous nuclear staining in DOX-treated MDA-MB-231 cells (Ghebeh et al., 2010; Rom-Jurek et al., 2018). In contrast, we found increased surface and cytoplasmic, but not nuclear, localization of PD-L1 in DOX-treated HCT116 cells (Figure 2C). Moreover, we observed a pronounced increase of surface fluorescence from anti-PD-L1 antibody staining upon 0.5 µM DOX treatment in HCT116 cells (Figure 2C), which is consistent with our western blot findings (Figures 1B and 1C). 

### 3.3. An inverse correlation was detected between PD-L1 expression with miR-140, but not with miR-34a, in DOX-treated HCT116 cells 

**Figure 3:**
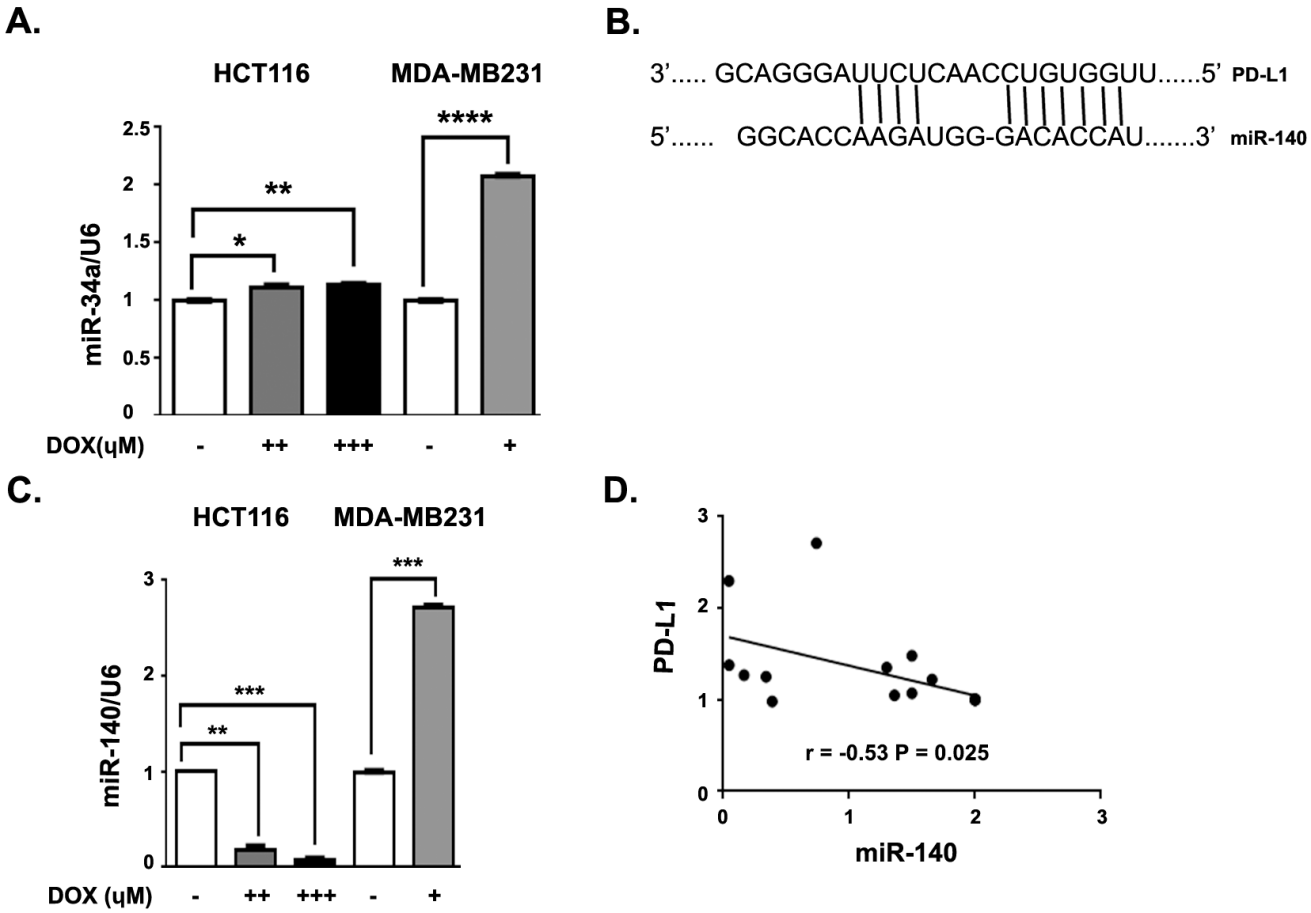
Doxorubicin regulates miR-140 expression in HCT116 and MDA-MB-231 cells in an opposite way. HCT116 and MDA- MB-231 cells were kept with various concentrations of DOX (for HCT116 cells (++) 0.5 ?M, (+++) 1.5 ?M; for MDA-MB-231 cells (+) 0.7 ?M) or without any treatment for 18 h. A) The expression of miR-34a in DOX-treated or nontreated HCT116 and MDA-MB-231 cells assessed by qRT-PCR. B) Schematic image of PD-L1 and miRNA-140 interaction. C) The expression of miR-140 in DOX-treated or nontreated HCT116 and MDA-MB-231 cells assessed by qRT-PCR. Data are normalized to U6 and are representative of 3 independent experiments. P values were calculated by unpaired t-test (**P < 0.01; ***P < 0.001. D) The correlations of PD-L1 with miR-140 were assessed. All values are expressed as means SEM. Data are normalized to U6. The data are representative of 3 independent experiments. P values are calculated by unpaired t-test (*P < 0.05, **P < 0.01; ***P < 0.001, ****P < 0.0001).

Recent reports indicated that PD-L1 is negatively regulated by miR-34a in a p53- dependent manner in Nutlin-3 (p53 stabilizer)-treated HCT116 cells (Cortez et al., 2015). While Nutlin-3 directly dissociates p53 from the ubiquitin ligase MDM2 by competitive binding, doxorubicin is thought to inhibit topoisomerase, result in DNA damage, and activate the damage response pathway that results in the phosphorylation of p53 and its dissociation from MDM2. To identify whether DOX treatment can also regulate miR-34a expression in HCT116 and MDA-MB-231 cells, we assessed the levels of this miRNA by qRT-PCR. We found a slight but significant increase of miR-34a expression in DOX-treated HCT116 cells in a dose-dependent manner compared with mock-treated cells (Figure 3A). In contrast, there was a significantly higher expression of miR-34a in DOX-treated MDA-MB-231 cells compared to HCT116 cells (Figure 3A). As we found that PD-L1 expression decreased in DOX-treated MDA-MB-231 cells (Figure 1) in which miR-34a expression was elevated (Figure 3A), it might be possible that PD-L1 is regulated by miR-34a in these cells. However, no inverse relationship between PD-L1 expression and miR-34a expression was detected in DOX-treated HCT116 cells. Therefore, another miRNA might be responsible for PD-L1 regulation in DOX-treated HCT116 cells.

In order to investigate potential miRNAs that might target PD-L1, we performed bioinformatics analysis using TargetScan (Xie et al., 2018). We observed that the PD-L1 mRNA’s 3’ UTR carries a putative miR-140 binding site at position 43–49 (Figure 3B). This putative binding site does not overlap with the previously identified binding site of miR-34a (position 932–938) on PD-L1 mRNA (Cortez et al., 2015). Next, we evaluated miR-140 expression in DOX-treated HCT116 and MDA-MB 231 cells. We determined that miR-140 expression was downregulated in DOX-treated HCT116 cells relative to the mock-treated control in a dose-dependent manner (Figure 3C). On the contrary, elevated miR-140 expression was detected in DOX-treated MDA-MB-231 cells. Also, our analysis revealed an inverse correlation between the miR-140 and PD-L1 expression in DOX-treated HCT116 cells (Figure 3D). This may imply that miR-140 has a role in regulation of PD-L1 in HCT116 cells. Thus, DOX treatment regulates expressions of PD-L1 and miRNA-140 in opposite ways in the breast and colorectal cell lines studied.

### 3.4. miR-140 partially regulates PD-L1 expression in HCT116 cells

**Figure 4:**
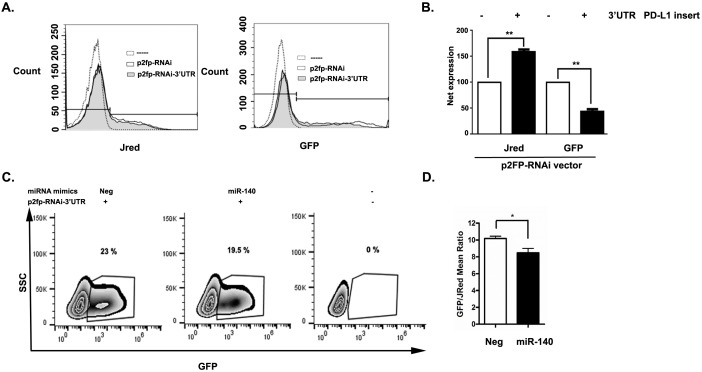
miR-140 binds and partially regulates PD-L1 in HCT116 cells. HCT116 cells were kept untreated (dotted histogram) or transfected either with p2FP-RNAi vector that included the 3’ UTR region of PD-L1 (p2FP-PD-L1 3’ UTR shaded histogram) or p2FPRNAi vector-only control (p2FP-RNAi-solid histogram). The p2FP-RNAi vector expresses two fluorescent proteins: TurboGFP and JRed. A) Representative histogram of JRed and GFP expression in untreated, p2FP-RNAi, and p2FP-PD-L1 3’ UTR. B) Net expression of JRed and GFP in groups shown in (A). C) HCT116 cells were kept untreated (-) or transfected with both p2FP-PD-L1 3’ UTR vector and with either control miRNA mimic (Neg) or miRNA-140 mimic (miRNA-140). Representative contour plot that depicts the percentage of GFP-positive cells. D) GFP/JRed mean ratio is shown for groups shown in (C). All values are expressed as means ± SEM. Significance of the data was calculated using one-way analysis of variance (ANOVA). P values are as follows: *P < 0.05; **P < 0.01.

Because our findings suggested a negative correlation between PD-L1 and miR-140, we assessed the presence of functional miRNAs in HCT116 cells, which bind to the 3’ UTR region of PD-L1. For that reason, we transfected HCT116 cells either with p2FP-RNAi plasmids that included the 3’ UTR region of PD-L1 (p2FP-PD-L1 3’ UTR) or p2FP-RNAi-only control plasmids. The p2FP-RNAi vector expresses two fluorescent proteins: TurboGFP and JRed. Because the 3’ UTR region of PD-L1 was cloned immediately following the TurboGFP gene, GFP fluorescence serves as a reporter of 3’ UTR miRNA activity, while JRed expression is used as a transfection normalization control. If HCT116 cells contain functional miRNAs targeting the PD-L1 3’ UTR, we expect decreased TurboGFP expression with no reduction in JRed expression. Indeed, we detected decreased TurboGFP expression in p2FP-PD-L1 3’ UTR HCT116 cells when compared to p2FP-RNAi plasmid-only transfected cells (Figures 4A and 4B). This result indicates that HCT116 cells contain functional miRNAs that regulate the PD-L1 3’ UTR. 

Next, we asked whether miR-140 would regulate PD-L1 expression in HCT116 cells. We transfected HCT116 cells with both p2FP-PD-L1 3’ UTR vectors and with either control miRNA mimic or miRNa-140 mimic RNA constructs. We determined that the miRNA-140 mimic slightly but significantly reduces gene expression dependent on the PD-L1 3’ UTR region (Figures 4C and 4D). Overall, these experiments demonstrate that miR-140 can regulate PD-L1 gene expression in HCT116 cells.

## 4. Discussion

Doxorubicin (DOX), a member of the anthracyclines, is a commonly used chemotherapeutic drug in the treatment of solid tumors such as lung and breast cancer. However, its usage in CRC treatment is limited due to chemoresistance to the drug at low doses and cardiotoxic effects at high doses of DOX (Ewer et al., 2015). 

Chemotherapy has been shown to have immune potentiating effects (Zitvogel et al., 2008). However, it is not known whether immune response to chemotherapeutic drugs is related to chemoresistance or chemosensitivity. It is reported that human tumors express a coinhibitory molecule, programmed cell death 1 ligand (PD-L1), at rates of 19%–92% (Sznol et al., 2013). PD-L1, expressed by cancer cells, binds to its receptors on T-cells and downregulates T-cell function and proliferation (Park et al., 2012). It is not clear how chemotherapeutic drugs affect the PD-1/PD-L1 axis in the tumor microenvironment. Earlier studies reported that DOX downregulates PD-L1 expression on the cell surface of MDA-MB-231 breast cancer cells (Ghebeh et al., 2010). Because the DOX effect on PD-L1 expression in CRC cells is not known, we investigated the expression level of PD-L1 on DOX treatment in HCT116 colon cancer cells. We found that PD-L1 expression increased positively in DOX-treated HCT116 colorectal cancer cells and decreased in MDA-MB-231 breast cancer cells both in RNA and protein levels (Figure 1). As PD-L1 expression is upregulated in DOX-treated CRC cells, it would be important to counteract PD-L1 using a blockade of PD-1/PD-L1 in DOX-treated CRC cells and evaluate whether this would increase the drug efficiency. It was reported that PD-L1 localized to the nucleus in DOX-treated MDA-MB-231 breast cancer cells (Ghebeh et al., 2010). In contrast, we demonstrate that PD-L1 is mainly localized on the surface and in the cytoplasm, but not in the nucleus of DOX-treated HCT116 cells (Figure 2). We surmise that the discrepancy of PD-L1 localization in HCT116 and MDA-MB-231 cells may be due to their distinct responses to DOX. 

It was recently reported that miR-34a directly targets and suppresses PD-L1 in breast cancer, lung cancer, colon cancer, and acute myeloid leukemia (Wang et al., 2015; Cortez et al., 2015). Concordantly, we found an increase of miR-34a both in DOX-treated HCT116 and MDA-MB-231 cell lines with a higher expression in the latter. However, there was only an inverse correlation between PD-L1 and miR-34a in DOX-treated MDA-MB-231 cells, not in HCT116 cells (Figure 3). Also, miR-140 was shown to target PD-L1 in lung and osteosarcoma cells (Ji et al., 2018; Xie et al., 2018). We found that miR-140 was upregulated in MDA-MB-231 cells and downregulated in HCT116 cells (Figure 3). We demonstrate a novel negative correlation between miR-140 and PD-L1 expression (r = –0.53, P < 0.025) in DOX-treated HCT116 colon cancer cells. Our findings are in line with a recently published manuscript showing an inverse correlation between miR-140 and PD-L1 in lung cancer (Xie et al., 2018). To our knowledge, this study is the first to show the effect of DOX on miR-34a and miR-140 and the specific mechanism of PD-L1 gene expression control in colon cancer cells. Additional studies are necessary to determine the role of miR-140 in PD-L1 regulation of DOX-treated HCT116 cells.

Taken together, our findings identify a distinct effect of DOX treatment on HCT116 colon cancer cells compared to MDA-MB-231 breast cancer cells in regard to PD-L1 and miR-140 expression. These findings illuminate a path toward future combined chemo- and immunotherapies that aim to increase the efficiency of colorectal cancer treatment. Such combination therapies could be formulated with lower doses of doxorubicin, combined with anti-PD-L1 antibody treatment, decreasing chemotherapy side effects.

## Authors’ contributions 

NN performed all experiments except miRNA mimic experiments and analyzed the data. NT performed miRNA mimic experiments. BE designed and supervised miRNA mimic experiments, edited the manuscript, and provided input. ASY designed and supervised the experiments and the data analysis and wrote the text. All authors have approved the final version of the manuscript.

## Acknowledgments

We would like to thank Tevriz Dilan Demir, Merve Aydın, and Sarah Mohammed Barakat for technical support. This study was supported by İstanbul Technical University, Department of Scientific Research Projects (ITÜ-BAP) (Project # 39555). The authors declare no conflict of interest.
